# Highly Pathogenic Avian Influenza A(H5N1) Virus RNA in Bovine Semen, California, USA, 2024

**DOI:** 10.3201/eid3205.251639

**Published:** 2026-05

**Authors:** Ailam Lim, Keith Poulsen, Leonardo C. Caserta, Lizheng Guan, Eryn Opgenorth, Maxwell P. Beal, Amie J. Eisfeld, Yoshihiro Kawaoka, Diego G. Diel

**Affiliations:** University of Wisconsin–Madison Wisconsin Veterinary Diagnostic Laboratory, Madison, Wisconsin, USA (A. Lim, K. Poulsen, E. Opgenorth); University of Wisconsin–Madison, Madison (A. Lim, K. Poulsen, L. Guan, A.J. Eisfeld, Y. Kawaoka); Cornell University Animal Health Diagnostic Center, College of Veterinary Medicine, Ithaca, New York, USA (L.C. Caserta, D.G. Diel); Mill Creek Veterinary Services, Visalia, California, USA (M.P. Beal); Dakota Dairy Health, Brookings, South Dakota, USA (M.P. Beal); University of Tokyo Institute of Medical Science, Tokyo, Japan (Y. Kawaoka); University of Tokyo Pandemic Preparedness, Infection and Advanced Research Center, Tokyo (Y. Kawaoka); Japan Institute for Health Security, National Institute of Global Health and Medicine, Tokyo (Y. Kawaoka)

**Keywords:** influenza, viruses, respiratory infections, highly pathogenic avian influenza, H5N1, biosecurity, California, United States

## Abstract

Since March 2024, highly pathogenic avian influenza (HPAI) A(H5N1) virus has infected dairy cattle in the United States, prompting concern about novel transmission routes. During an outbreak in California, HPAI H5N1 RNA was detected in an asymptomatic bull’s semen. Although infectious virus was not isolated, semen-associated transmission risks and biosecurity practices remain a concern.

Since March 2024, detection of clade 2.3.4.4b highly pathogenic avian influenza (HPAI) A(H5N1) in US dairy cattle has raised concerns about the virus’s ability for cross-species transmission, adaptation to mammals, and novel transmission routes, including milk (*1*,*2*). Multiple pathogenic viruses are transmitted in bovine semen, and detection of HPAI in turkey semen has prompted questions about the potential role of HPAI transmission in bovine semen (*3*,*4*). Shedding of HPAI H5N1 in bovine semen could result in silent viral spread within herds and across geographic regions through artificial insemination. Although clinical HPAI disease has been reported in female calves and pregnant animals, reports of diseased bulls in dairy farms or beef cattle are lacking. Many questions about the pathophysiology of HPAI H5N1 in US dairy herds remain unanswered, but movement of lactating cows is a recognized risk factor for interstate disease spread. In this diagnostic study, we sought evidence of HPAI H5N1 shed through semen in natural breeding bulls on an HPAI H5N1–affected dairy farm in California.

The HPAI H5N1 genotype B3.13 outbreak in California began in August 2024 and likely resulted from the interstate movement of infected cows, which led to the rapid spread of the virus within the state. In October 2024, infection in a 4,500-head Holstein dairy was detected by reverse transcription PCR (RT-PCR) for H5N1 RNA in bulk tank milk samples. Clinical signs in lactating dairy cows consisted of decreased milk production, mastitis, lethargy, dehydration, anorexia, and pyrexia (>104.0°F). The herd experienced a 60% illness rate over 3 weeks. About 4 weeks after detection, the herd veterinarian collected diagnostic samples from 3 individual 3-year-old Holstein bulls; samples consisted of deep nasal swabs, preputial scrapings, preejaculate seminal fluid, semen, and serum samples. Those samples were sent to the Wisconsin Veterinary Diagnostic Laboratory (Madison, WI, USA) for testing. Subjectively, the semen appeared to have low sperm concentration and volume, although total sperm count was not measured. The poor semen quality was likely because of timing and suboptimal sampling conditions; the bulls had been comingled with cows the same day for breeding.

Initially, we examined the samples for influenza A virus (IAV) using various methods (Table; Appendix). Deep nasal swabs, preputial scrapes, preejaculate seminal fluids, and semen samples were tested using multiple IAV Matrix RT-PCRs (*5*,*6*). IAV RNA was detected at a low level in semen from bull 1 by 3 different PCR assays, but bull 2 and bull 3 tested negative. No detection was reported in any other samples. The IAV RNA detection in bull 1 was confirmed with an additional RNA extraction to rule out laboratory contamination. The IAV strain was further identified as HPAI using the H5N1 2.3.4.4b lineage subtyping RT-PCR.

**Table:** Influenza A virus testing results for samples collected from 3 bulls in HPAI H5N1–infected farm in study of HPAI H5N1 virus RNA in bovine semen, California, USA, 2024*

Bull no.	Specimen type	IAV ELISA result (sample/positive control ratio)	IAV RT-PCR result (Ct)†	H5 RT-PCR result (Ct)†	Clade 2.3.4.4b RT-PCR result (Ct)†	Virus isolation
Bull 1	Serum	Negative (0.620)	NP	NP	NP	Negative
	Deep nasal swabs	NP	Negative	NP	NP	Negative
	Preputial scrape	NP	Negative	NP	NP	Negative
	Preejaculate	NP	Negative	NP	NP	Negative
	Semen	NP	Detected:‡ detected (38.2/39.5);§ negative/detected (39.1);¶ detected (37.9/38.9)#	Negative	Detected (39.6)	Negative
					
					
					
Bull 2	Serum	Negative (1.000)	NP	NP	NP	NP
	Deep nasal swabs	NP	Negative	NP	NP	NP
	Preputial scrape	NP	Negative	NP	NP	NP
	Preejaculate	NP	Negative	NP	NP	NP
	Semen	NP	Negative	NP	NP	NP
Bull 3	Serum	Negative (0.880)	NP	NP	NP	NP
	Deep nasal swabs	NP	Negative	NP	NP	NP
	Preputial scrape	NP	Negative	NP	NP	NP
	Preejaculate and semen	NP	Negative	NP	NP	NP

*Ct, cycle threshold; HPAI, highly pathogenic avian influenza; IAV, influenza A virus; NP, not performed; RT-PCR, reverse transcription PCR. †Assay Ct cutoff value was 40. ‡Sample was subjected to further sequencing analyses. §Wisconsin Veterinary Diagnostic Laboratory IAV assay, tested in duplicate. ¶National Animal Health Laboratory Network IAV Matrix assay, tested in duplicate. #Modified National Animal Health Laboratory Network IAV Matrix assay, tested in duplicate.

Targeted IAV sequencing confirmed the presence of H5N1 virus and yielded a partial H5N1 genome (Appendix Table 1), deposited into GISAID (https://www.gisaid.org; accession no. EPI_ISL_20206713). Attempts to assemble the full genome were unsuccessful because of the low viral load in the semen. Phylogenetic analysis of the partial concatenated genome sequences indicated that the viral RNA in bull 1 clustered within predominantly bovine-derived B3.13 genotype sequences and was most closely related to a sample collected from a dairy farm worker in California during the same period (Figure) (*7*).

**Figure:**
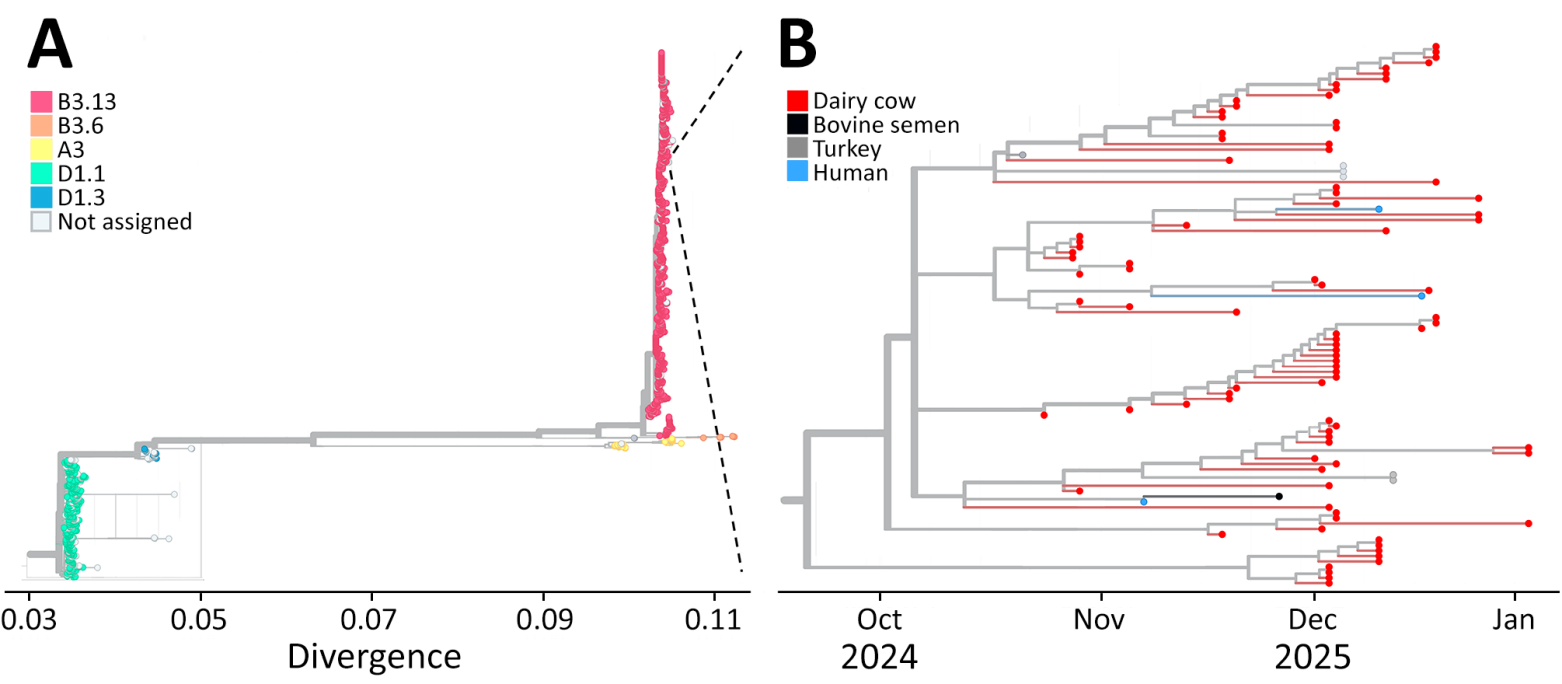
Phylogenetic analysis of partial concatenated genome sequences confirming detection of highly pathogenic avian influenza A(H5N1) virus genotype B3.13 from bovine semen, California, USA, 2024. A) Tree showing broader phylogeny of H5N1 virus genotypes. B) Timescale tree showing closer examination of the virus from bull 1 semen (bovine semen, black line) and closely related virus sequences.

We processed all samples from bull 1 for virus isolation to attempt recovery of an isolate. Serially diluted samples inoculated into 10-day-old specific-pathogen–free embryonated chicken eggs or Madin-Darby canine kidney cells showed no embryo death or cytopathic effect. We confirmed all samples as negative by hemagglutination assay.

The serum samples from all bulls tested negative by antigen-based influenza A ELISA (*8*). However, the sample/positive control ratio for bull 1 was 0.620, close to the validated 0.5 assay cutoff, potentially indicating seroconversion.

Because of the limited semen volume available for analysis, we did not perform further confirmation testing at the national reference laboratory. We requested additional samples several months later for convalescent testing, but bull 1 had been culled from the herd. The significance of identifying HPAI H5N1 in bovine semen remains uncertain. The virus could have been actively shed in semen, or the ejaculate could have been contaminated during collection. Although detecting RNA does not confirm the presence of infectious virus, this finding warrants further investigation into whether HPAI H5N1 can be shed in semen and raises questions about farm biosecurity amid the ongoing outbreak. High viral load in the environment during a herd outbreak was well documented (S. Lakdawala et al., unpub. data, https://www.biorxiv.org/content/10.1101/2025.07.31.666798v3; C. Stenkamp-Strahm et al., unpub. data, https://www.medrxiv.org/content/10.1101/2025.09.03.25335023v1). Good biosecurity measures are essential to prevent infections and, if infection occurs, slow disease spread on the farm.

In conclusion, further research and risk assessments are needed to determine tissue tropism of HPAI H5N1 in reproductive organs and whether naturally infected bulls shed virus in semen, and, if so, evaluate the risk for disease spread on dairy farms and with artificial insemination programs. Repetition and confirmation of these findings would have implications for natural breeding and biosecurity for artificial insemination collection centers, suggesting the need for increased caution in preventing silent intraherd spread.

Appendix Additional information about highly pathogenic avian influenza A(H5N1) viral RNA in bovine semen, California, USA, 2024
