# Supplementary material for: Highly Pathogenic Avian Influenza A(H5N1) Virus RNA in Bovine Semen, California, USA, 2024
Source: Emerg Infect Dis. 2026 May;32(5):824–6. doi: 10.3201/eid3205.251639 (PMC13175099; doi:10.3201/eid3205.251639)
Supplement: Appendix — Additional information about highly pathogenic avian influenza A(H5N1) viral RNA in bovine semen, California, USA, 2024 [file 25-1639-Techapp-s1.pdf]

Article DOI: https://doi.org/10.3201/eid3205.251639

EID cannot ensure accessibility for supplementary materials supplied by authors. Readers who have difficulty accessing supplementary content should contact the authors for assistance.

Highly Pathogenic Avian Influenza A(H5N1) Virus RNA in Bovine Semen, California, USA, 2024

Appendix

Materials and Methods

Sample collection and preparation. Due to movement restrictions in effect during the HPAI outbreak in cattle, sampling of the bulls was conducted on-farm. Given the lack of facilities to handle the 2500–3000 lb full-grown, commercial, Holstein bulls, a mobile chute and temporary fencing were set up to corral the bulls. Once in the chute, the bulls' size posed a significant challenge to proper restraint. Two of the five bulls were excluded from sampling due to safety concerns for the bulls, the workers and the attending veterinarian.

Serum was collected in no-additive red-top tubes from the remaining three bulls. Deep nasal swabs and preputial scrapings from all three bulls were collected into 3 ml brain heart infusion (BHI) media. Pre-ejaculate seminal fluid and semen were collected separately from Bull-1 and Bull-2, while a mixture of pre-ejaculate and semen was collected from Bull-3. Semen was collected using a standard bull breeding soundness examination protocol. The prepuce and sheath were clipped and cleansed before collection. Accessory sex glands were stimulated via rectal palpation to obtain a pre-ejaculate sample. Semen was subsequently collected using an electro ejaculator. Procedure was performed by the attending veterinarian with gloved hands.

It was an onerous task to move the untamed bulls through the chutes for sample collection, the entire process took a veterinarian and 5 dairy staff ≈3 hours to complete. All animals were returned to their respective pens without complication.

Samples were shipped on ice packs to Wisconsin Veterinary Diagnostic Laboratory. Upon arrival, serum was separated from clots and store at 4°C until testing. Preputial scrape was pelleted by centrifugation at 12,000 xg, supernatant was transferred into a new tube and pellet was resuspend in 500 µL of 1x phosphate buffered saline (PBS) for storage at −80°C. Pre-ejaculate seminal fluid and semen were stored at −80°C until tested.

DNA extraction and RT-PCR assays. Samples were extracted at Wisconsin Veterinary Diagnostic Laboratory using the IndiMag Pathogen Kit (Indical BIOSCIENCE, Lepzig, Germany). A 200 µL volume input of media for deep nasal swabs or preputial scrapes were used, while 100 µL of resuspended preputial scrape pellets, pre-ejaculate or semen were diluted 1:2 in 1x PBS for extraction (1); Assay specific Internal Positive Controls were added to the lysis solution. The extraction process was conducted according to the manufacturer's instructions using the KingFisher Flex extractor (Thermo Fisher Scientific, Waltham, MA). Eluted RNA was used for RT-PCR evaluation or stored in at −80°C.

The RNA extracted was evaluated using three IAV RT-PCR assays. The Wisconsin Veterinary Diagnostic Laboratory in-house IAV assay was conducted as previously published (1); the National Animal Health Laboratories Network (NAHLN) IAV Matrix RT-PCR assay was conducted according to the NAHLN standard protocol NVSL-SOP-0068 (*2*) using the AgPath-ID One-Step RT-PCR Reagents (Thermo Fisher Scientific, Waltham, MA; while the Modified NAHLN IAV Matrix assay was an approved version of the of the NAHLN assay using the IndiMix JOE master mix (Indical BIOSCIENCE, Lepzig, Germany) as alternative reagent in a 20 µl reaction (A. Hach et al., unpub. data, https://www.preprints.org/manuscript/202603.0089). Upon positive detection on the IAV assays, subtyping RT-PCR assays for the H5 subtype and the H5N1 2.3.4.4b lineage were carried out according to protocols in the NVSL-SOP-0068. The NAHLN-approved protocols are available online through the APHIS-Approved Testing Laboratory Documents (https://www.aphis.usda.gov/animal_health/lab_info_ services/downloads/ApprovedSOPList.pdf). The NAHLN program office controls the distribution of protocols; these can be requested by contacting nvsl.mastercontrol@usda.gov.

Targeted influenza A sequencing and bioinformatic analyses. The semen sample was shipped frozen to the Cornell Animal Health Diagnostic Center for sequencing. Sample was

diluted 1:2 in PBS, and 200 µl of this dilution was used for extraction with the IndiMag Pathogen Kit (Indical BIOSCIENCE, Lepzig, Germany) on the KingFisher Flex extractor (Thermo Fisher Scientific, Waltham, MA), as well as manually extracted using the QIAamp MinElute Virus Spin Kit (Qiagen Science, Germantown, MD). A tiled-amplicon approach was used to amplify all segments of H5N1 viruses using two primer pools (primer sequences available upon request). Sequencing libraries were prepared using the Native Barcoding Kit EXP-NBD196 and the Ligation Sequencing Kit SQK-LSK109 (Nanopore Technologies, Oxford, United Kingdom), and sequenced on a FLO-MIN106 MinION flow cell (R9.4.1) using the GridION platform (Nanopore Technologies, Oxford, United Kingdom). Quality-filtered and primer-trimmed reads were aligned to a reference genome downloaded from GenBank using Minialign software (v0.4.4; https://github.com/ocxtal/minialign). Consensus sequences were generated using Medaka (v1.4.3) with the medaka_haploid_variant and medaka_consensus programs for polishing (https://github.com/nanoporetech/medaka). The dataset used for analysis consisted of HPAI H5N1 genomes from samples collected between August and December 2024 in North America, downloaded from the GISAID EpiFlu database. Phylogenomic analyses were performed using Nextstrain (*3*), with a modification to input a maximum-likelihood phylogenetic tree of concatenated genomes inferred using IQ-TREE (*4*) with an edge-linked partition model and 1,000 bootstrap replicates.

Viral Isolation. All the samples from Bull-1 were transported frozen to the University of Wisconsin—Madison Influenza Research Institute for viral isolation. Specific-pathogen-free (SPF) embryonated chicken eggs (VALO BioMedia, Adel, IA) were incubated at 37°C with appropriate humidity. Ten-day-old eggs were candled to verify embryo viability before inoculation. Madin-Darby canine kidney (MDCK) cells were grown in Eagle's minimal essential medium (MEM) containing 5% newborn calf serum and antibiotics at 37°C in a humidified atmosphere of 5% CO_2. Cells were routinely monitored for mycoplasma contamination.

After thawing, liquid samples (deep nasal swabs, preputial wash, pre-ejaculate, and semen) were used as-is. The thawed preputial cell pellet was resuspended in 0.5 ml of phosphate-buffered saline (PBS). All samples were then serially diluted (10^{-1} through 10^{-5}) in PBS and used to inoculate SPF eggs (10° through 10^{-2} dilutions, 100 µl per egg, 2 eggs per dilution) or MDCK cells in 6-well plates (10° through 10^{-5} dilutions, 500 µl per well, 1 well per dilution). For MDCK cells, inoculums were removed after 1 hour of incubation at 37°C, cells were washed

once with virus growth medium (MEM containing 0.3% bovine serum albumin and 0.6 µg/ml of L-(tosylamido-2-phenyl) ethyl chloromethyl ketone (TPCK)-treated trypsin), and then covered with virus growth medium. Eggs were incubated as described above for 3 days, candled to assess viability, and then killed by incubation at 4°C overnight. MDCK cells were incubated as described above for 5 days and observed daily by microscopy to assess cytopathic effects. No blind passage was performed.

Hemagglutination assays. Allantoic fluids from eggs or MDCK cell culture supernatants were subjected to hemagglutination (HA) assays according to standard methods. Briefly, allantoic fluids or MDCK supernatants were 2-fold serially diluted and mixed with 0.5% turkey red blood cells, followed by assessment for agglutination.

Bovine influenza A ELISA. Sera were tested at 1:5 dilution using the IDEXX Al MultiS-Screen Enzyme-Linked lmmunosorbent Assay (IDEXX, Westbrook, ME, USA). The test was performed per manufacturer's instructions, and interpretation per the NAHLN protocol NVSL-SOP-1255 (*5*) where sample over positive control (S/P) ratio ≥0.5 are considered as indicating absence of influenza A specific antibodies.

Biosafety. Attempted virus isolation from bull samples was carried out in a Biosafety Level 3 (BSL-3) containment laboratory at the Influenza Research Institute at the University of Wisconsin-Madison, which is approved by the Federal Select Agent Program for studies with highly pathogenic avian influenza viruses. The University of Wisconsin-Madison Institutional Contact for Dual Use Research reviewed this manuscript and confirmed that the studies described herein do not meet the criteria of Dual Use Research of Concern (DURC).

References

1. Zimmerman A, Vandenburg-Carroll A, Marthaler DG, Lim A. Optimizing Nucleic Acid Extraction from Extended Bovine Semen for Endemic and High-Consequence Pathogens. Animals (Basel). 2025;15:3411. PubMed https://doi.org/10.3390/ani15233411

2. NVSL-Supervisor-DVL-MOLECULAR Section Head. Real-time RT-PCR detection of influenza A and avian paramyxovirus type-1. Riverdale Park (MD): Animal and Plant Health Inspection Service; 2024 Sep 30. Document no. NVSL-SOP-0068, revision 6. 3. Hadfield J, Megill C, Bell SM, Huddleston J, Potter B, Callender C, et al. Nextstrain: real-time tracking of pathogen

evolution. Bioinformatics. 2018;34:4121–3. PubMed https://doi.org/10.1093/bioinformatics/bty407

4. Minh BQ, Schmidt HA, Chernomor O, Schrempf D, Woodhams MD, von Haeseler A, et al. IQ-TREE 2: new models and efficient methods for phylogenetic inference in the genomic era. Mol Biol Evol. 2020;37:1530–4. PubMed https://doi.org/10.1093/molbev/msaa015

5. Posey E. Use of IDEXX influenza A ELISA for antibody detection in bovine-origin mik and serum. Riverdale Park (MD): Animal and Plant Health Inspection Service; 2024 Sep 6. Document no. NVSL-SOP-1255, revision 3.

Appendix Table. Genome coverage of the eight influenza A segments for sample Bull 1 generated using a tiled-amplicon approach on a long-read sequencing technology using MinION flow cell (R9.4.1) on the Oxford Nanopore GridION platform. The sequence generated was deposited in GISAID (accession number EPI_ISL_20206713).

Segment #	Segment Name	No. reads assembled to reference	Segment % Coverage
1	Polymerase basic 2 (PB2)	35992	61
2	Polymerase basic 1 (PB1)	2431	19
3	Polymerase acidic (PA)	5972	47
4	Hemagglutinin (HA)	6342	33
5	Nucleocapsid (NP)	7717	47
6	Neuraminidase (NA)	11616	72
7	Matrix (M)	1019	44
8	Nonstructural (NS)	10182	60
